# Editorial: Turning fishery by-products into nutritional gold: strategies and innovations

**DOI:** 10.3389/fnut.2026.1987874

**Published:** 2026-09-28

**Authors:** Daniela Lorena Lamas, Vijay Kumar Reddy Surasani

**Affiliations:** 1 Institute of Marine and Coastal Research IIMyC-CONICET, Mar del Plata, Buenos Aires, Argentina; 2 Department of Fish Processing Technology, College of Fisheries, GADVASU, Ludhiana, Punjab, India

**Keywords:** aquatic food systems, biofertiiizers, circular bioeconomi, fishery by-product, functional food, protein hydrolysate, sustainable packaging, waste valorization

## 1 From ocean losses to circular treasures: a paradigmatic shift

Every year, millions of metric tons of nutrient-dense aquatic biomass never reach the human plate. Instead, skins, bones, viscera, and processing trimmings are discarded or relegated to low-value applications. This represents not only an environmental liability but a profound loss for global food security. In an era dictated by resource scarcity, climate urgency, and hidden hunger, viewing aquatic by-products merely as “waste” is an obsolete luxury. Moving toward a sustainable circular bioeconomy demands a radical shift in perspective: converting these side-streams into “nutritional gold.” Aquatic processing residues are rich reservoirs of high-quality proteins, essential long-chain omega-3 fatty acids, micronutrients, and bioactive peptides. The fundamental challenge—and opportunity—lies in deploying innovative, integrated, and scalable strategies to recover and transform these compounds into technological, agricultural, and nutritional solutions.

This Research Topic gathers four pioneering contributions that collectively map out the multi-dimensional pathways needed to bridge the gap between seafood waste and sustainable resource stewardship.

## 2 The four pillars of valorization: bridging science, food security, and materials

### 2.1 Pillar I: nourishing the vulnerable through optimized traditional processing

The journey toward zero loss begins at the community level, where traditional fish processing dictates local nutrition. Ahern et al. provided a compelling demonstration of how optimizing capture-fishery post-harvest processing in Zambia directly addresses malnutrition in critical demographic windows. By comprehensively evaluating the nutrient profiles of inland fish products, the authors revealed that improved smoking technologies and fish powders dramatically preserve vital micronutrients (Vitamins A and E) and omega-3 fatty acids (DHA meeting up to 33% of daily requirements for infants and lactating mothers) compared to traditional methods. This work underscores a vital truth: valorization is not solely about high-tech extractions; it is fundamentally about preserving life-sustaining nutrients to empower human health during the first 1,000 days of life.

### 2.2 Pillar II: beyond food—marine proteins as green biomaterials

Valorization extends far beyond edible applications, offering solutions to global plastic pollution. Mishra et al. tackled the synthetic packaging crisis by harnessing myofibrillar proteins extracted from Basa fish (*Pangasius bocourti*) processing waste to construct biodegradable food packaging films. By fine-tuning structural parameters—specifically demonstrating that a 1% protein concentrate at pH 2.8 optimizes mechanical resilience and barrier properties—the authors illustrated how structural proteins recovered from these fish side-streams can replace persistent petroleum-based polymers. This contribution elevates fishery by-products from nutritional assets to versatile biopolymers capable of protecting the very food systems from which they originate.

### 2.3 Pillar III: ocean-to-land synergies—closing the agricultural nutrient loop

True circularity transcends domain boundaries, connecting aquatic systems with terrestrial agriculture. Solis et al. demonstrated this inter-systemic synergy by converting processing by-products from *Brama australis* and *Merluccius australis* into bio-based agricultural inputs via combined enzymatic and microbial bioprocessing. The resulting hydrolysates, rich in bioavailable nitrogen and bioactive peptides, significantly enhanced wheat crop growth and biomass yield when applied as biofertilizers. This work exemplifies how nutrients salvaged from ocean depths can rejuvenate terrestrial soils, closing nutrient loops across marine and agrarian ecosystems.

### 2.4 Pillar IV: cross-sectoral innovation—creating next-generation hybrid foods

At the frontier of functional food design lies the integration of distinct agro-industrial waste streams into single, highly nutritious matrices. Lamas and Aguiló-Aguayo pushed the boundaries of circular food design by formulating hybrid oat-okara functional spreads enriched with upcycled salmon protein hydrolysates and fish oils. By harmonizing side-streams from both plant-based beverage production (okara) and aquatic processing, this study offers a proof-of-concept for next-generation, cross-sectoral functional foods. The resulting hybrid spread demonstrates that marine bioactive compounds can seamlessly elevate plant matrices, delivering tailored nutritional profiles for specialized human health needs.

## 3 A unified vision for a sustainable tomorrow

As conceptualized in our overarching framework (Figure 1), these four contributions should not be viewed in isolation. Together, they represent a continuum of solutions ranging from frontline nutrition and green packaging to agro-ecological bioinputs and advanced functional food design.

**Figure 1:**
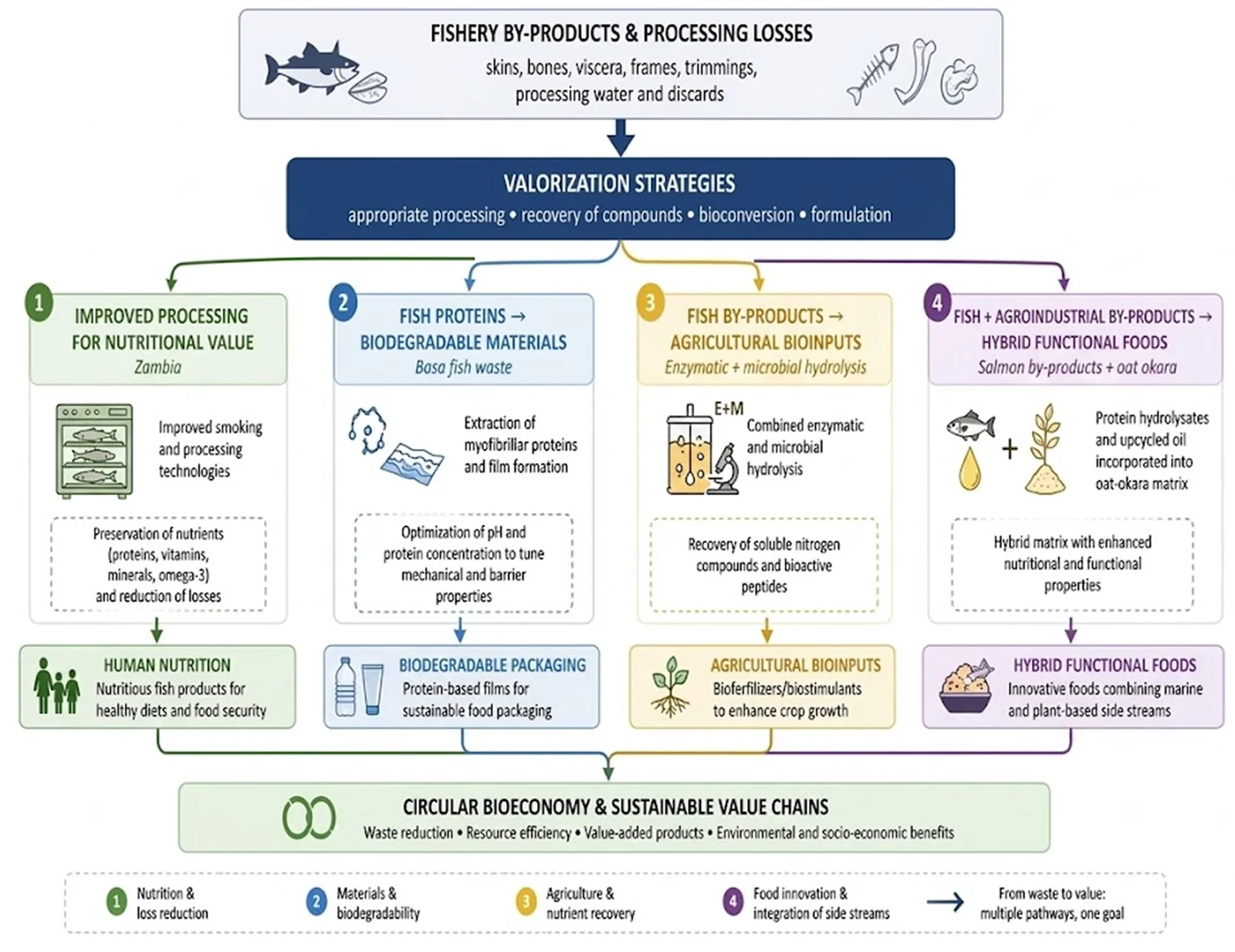
Strategic pathways for the valorization of fishery and aquatic processing by-products within a circular bioeconomy framework. The overarching scheme illustrates the transformation of processing residues (skins, bones, viscera, trimmings, and frames) into high-value applications across four thematic pillars: (1) Preservation of essential nutrients (proteins, vitamins, minerals, omega-3) through improved processing to enhance human nutrition in vulnerable populations (Zambia); (2) Recovery of myofibrillar proteins for the development of biodegradable food packaging materials (Basa fish waste); (3) Combined enzymatic and microbial bioprocessing to recover bioavailable nitrogen and bioactive compounds for agricultural bioinputs (*Brama australis* and *Merluccius australis*); and (4) Integration of marine hydrolysates and upcycled oils with plant-based side-streams (oat okara) for the design of next-generation hybrid functional foods (Salmon by-products). Together, these approaches connect aquatic and terrestrial value chains, promoting resource efficiency, waste reduction, and sustainable development goals (SDGs 2, 12, and 14).

To move these strategies from laboratory breakthroughs to scalable industrial paradigms, future research must focus on:

1. Green and scalable extractions: implementing low-energy, solvent-free bioprocesses that preserve molecular integrity while lowering economic hurdles.
2. Sensory and regulatory harmony: overcoming flavor profiles and establishing clear safety protocols to ensure rapid consumer acceptance and market integration.
3. Cross-industry alliances: fostering interdisciplinary partnerships between fish and aquatic scientists, food engineers, agronomists, and packaging industries.

Ultimately, these studies align directly with the United Nations Sustainable Development Goals: eradicating hunger (SDG 2), driving responsible consumption and production (SDG 12), and conserving marine resources through sustainable utilization (SDG 14).

We hope this collection inspires researchers, policymakers, and industry leaders to stop seeing fishery by-products as a burden to dispose of, and to start recognizing them for what they truly are: a golden opportunity to nourish and sustain our planet.

